# Gut Microbiota Differences in Wild Asian Elephants From Pu'er, China

**DOI:** 10.1002/ece3.74333

**Published:** 2026-09-08

**Authors:** Zheng Jing, Zhou Jiuxuan, Liu Xia, Wang Binghui, Wang Yixuan, Cao Hui, Lu Tihuo

**Affiliations:** ^1^ College of Forestry Southwest Forestry University Kunming China; ^2^ Yunnan Academy of Forestry and Grassland Kunming China; ^3^ School of Public Health & Yunnan Province Key Laboratory of Public Health and Biosafety Kunming Medical University Kunming China

**Keywords:** 16S rRNA, gut Microbiota, habitat differences, wild Asian elephants

## Abstract

The gut microbiota of Asian elephants plays a critical role in their health and habitat adaptation. This study compared the gut microbiota of two wild Asian elephant populations in Jiangcheng (JC) and Liushun (LS), Yunnan, China. Significant differences in microbial structure, diversity, and function were found. Across samples, 24 phyla, 48 classes, 98 orders, 164 families, 288 genera, and 351 species were annotated. JC and LS shared 1897 OTUs, with 3890 and 3789 unique OTUs, respectively. Both groups were dominated by Firmicutes, Bacteroidetes, and Verrucomicrobia, but Proteobacteria was uniquely dominant in LS. At the family level, Flavobacteriaceae contributed most to JC differences (LDA≈4.2), while Bacteroidaceae (LDA≈4.0) drove LS differences. Alpha diversity showed no significant difference in Chao1, but Shannon and Simpson indices were significantly higher in JC than in LS (*p* < 0.05). Functional prediction revealed JC was enriched in genetic information processing and energy metabolism, whereas LS showed higher proportions of environmental adaptation and material metabolism pathways. These differences likely reflect food resources and environmental factors, indicating gut microbiota plasticity in response to heterogeneous habitats. The findings provide valuable insights for habitat quality assessment and gut microecological conservation of Asian elephants.

## Graphical abstract

This study compared the gut microbiota of two wild Asian elephant populations in Jiangcheng (JC) and Liushun (LS), Yunnan, China. Significant differences in microbial structure, diversity, and function were found. Across samples, 24 phyla, 48 classes, 98 orders, 164 families, 288 genera, and 351 species were annotated. JC and LS shared 1897 OTUs, with 3890 and 3789 unique OTUs, respectively. Both groups were dominated by Firmicutes, Bacteroidetes, and Verrucomicrobia, but Proteobacteria was uniquely dominant in LS. At the family level, Flavobacteriaceae contributed most to JC differences (LDA≈4.2), while Bacteroidaceae (LDA≈4.0) drove LS differences. Alpha diversity showed no significant difference in Chao1, but Shannon and Simpson indices were significantly higher in JC than LS (*p* < 0.05). Functional prediction revealed JC was enriched in genetic information processing and energy metabolism, whereas LS showed higher proportions of environmental adaptation and material metabolism pathways. These differences likely reflect food resources and environmental factors, indicating gut microbiota plasticity in response to heterogeneous habitats. The findings provide valuable insights for habitat quality assessment and gut microecological conservation of Asian elephants.

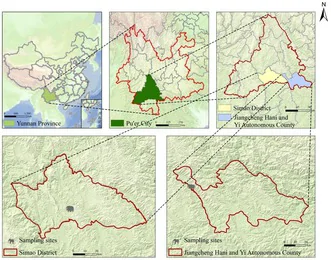

## 1 Introduction

The Asian elephant ( *Elephas maximus* ) is the largest extant terrestrial mammal in Asia and a typical flagship species, playing an important role in maintaining the structure and function of forest ecosystems. In China, it is listed as a first‐class national protected wildlife species and assessed as endangered by the International Union for Conservation of Nature (IUCN) (Wang et al. 2021). Historically, Asian elephants were widely distributed across South and Southeast Asia (Olivier 1978); however, due to habitat loss and human activities, their range has significantly contracted, and they now occur only in scattered populations across 13 countries, including China (Li 1998). Against the background of global population declines, the Asian elephant population in China has shown a recovery, increasing from approximately 150 individuals at the end of the 20th century to over 300 individuals by 2025, with a particularly notable increase in Yunnan Province (Li 2017).

Asian elephants are hindgut‐fermenting herbivores. Their own genome does not encode cellulose‐degrading enzyme systems, and their utilization of plant fiber relies on gut microbial fermentation (Imlberger et al. 2014; Chong et al. 2019). The gut microbiota plays a critical role in nutrient breakdown, energy acquisition, and host health maintenance in herbivorous mammals. Studies have shown that the gut microbiota of elephants is primarily composed of Firmicutes, Bacteroidetes, and Proteobacteria, with compositional differences observed across species and habitats, and these differences are closely related to diet composition and environmental factors (Budd et al. 2020). In the African savanna elephant ( *Loxodonta africana* ) and the African forest elephant (*Loxodonta cyrictis*), the most common phyla in the gut are Firmicutes, Protebacteria, and Bacteroidetes. The gut microbiota of the African savanna elephant is dominated by Firmicutes, whereas that of the African forest elephant is dominated by Protebacteria, a difference attributed to dietary variations resulting from their distinct habitats (Budd et al. 2020). The gut microbiota also differs between captive and wild Asian elephants. A study on three wild Asian elephants at the Wild Elephant Valley in Xishuangbanna found that the most abundant phylum was Firmicutes, followed by Bacteroidetes, Proteobacteria, Fibrobacteres, Spirochaetes, and Actinobacteria (Klinhom et al. 2025); in contrast, another study on captive Asian elephants reported that the dominant gut microbiota consisted mainly of Firmicutes, Bacteroidetes, Proteobacteria, and Spirochaetes (Klinhom et al. 2025). Studies on individual wild Asian elephants have shown a high abundance of Firmicutes and cellulose‐degrading enzymes in the gut, indicating a strong capacity for lignocellulose degradation (Zhang et al. 2019; Lin et al. 2016); metagenomic analyses further revealed that the gut microbiota of wild Asian elephants is enriched with functional enzymes involved in the degradation of hemicellulose and complex polysaccharides (Klinhom et al. 2025). Research has found that the transition between wild and captive states can lead to significant changes in gut microbiota composition, which may affect the host's ability to adapt to the environment (Chong et al. 2019). Due to the relatively monotonous diet structure of captive individuals, their gut microbiota diversity and functional potential are reduced (Mohamed et al. 2021; Wang, Wang, Zhou, et al. 2024). Sarisa Klinhom and colleagues collected fecal samples from 18 Asian elephants in Chiang Mai, Thailand, and performed sequencing analysis. They found that the most abundant phylum in healthy Asian elephants was Firmicutes, followed by Bacteroidetes. Compared to healthy individuals, elephants suffering from colic and diarrhea exhibited reduced levels of Bacteroidetes. Healthy Asian elephants had a relatively even composition of gut bacterial communities, whereas those with gastrointestinal diseases showed marked changes in the abundance of specific bacterial taxa and significantly lower alpha diversity than healthy individuals, indicating that gastrointestinal diseases significantly affect the gut microbiota composition of Asian elephants (Klinhom et al. 2025).

Wild Asian elephants exhibit strict selectivity for habitat factors such as food type and quantity, habitat area, concealment conditions, water availability, and temperature. They primarily inhabit tropical rainforests, seasonal rainforests, and forested ravines and thickets below an altitude of 1000 m (Tan and Yu 2015). Regarding Simao District specifically, land use changes are mainly characterized by a reduction in forest land, cultivated land, and grassland, which have been converted into other land types, along with an increase in plantation land, construction land, and water areas. Among these, plantation land showed an average annual growth rate of 3.9%. Changes in forest land use are the primary cause of the ongoing reduction and fragmentation of suitable habitats for Asian elephants. Currently, suitable habitat area accounts for only 14%, indicating severe habitat fragmentation (Jin et al. 2023). Further studies have found that during the dry season, Asian elephants in Simao primarily consume 19 species of wild plants and 11 species of crops and economic trees. The main factors influencing their habitat use include food resources, concealment conditions, and human disturbance (Wang and Zhang 2001).

This study focuses on wild Asian elephants from Jiangcheng and Liushun in Pu'er, Yunnan Province. Using 16S rRNA high‐throughput sequencing technology, we analyzed the composition, diversity, and functional differences of their gut microbiota. The main objectives were to investigate whether there are significant differences in gut microbiota structure between wild Asian elephants inhabiting different habitat conditions, to characterize regional variations in microbial diversity and dominant taxa, and to further elucidate the influence of gut microbial functions on ecological environmental differences. The results of this study contribute to understanding the adaptive responses of gut microbiota in herbivorous mammals to heterogeneous habitats, and provide scientific references for habitat management, ex situ conservation, and optimization of semi‐natural rearing models for Asian elephants. Therefore, to investigate the adaptive responses of gut microbiota to heterogeneous habitats, the Jiangcheng (JC) and Liushun (LS) sites in Pu'er were selected for this comparative study. These two subpopulations represent contrasting habitat conditions within the same regional context: JC is characterized by relatively intact tropical seasonal evergreen broadleaf forest with stable food resources, whereas LS experiences higher anthropogenic pressure from agricultural expansion and infrastructure development, leading to more fragmented habitat and fluctuating food availability. This paired‐site design allows for a controlled comparison to elucidate the influence of habitat quality on gut microbial ecology.

## 2 Study Area

**FIGURE 1:**
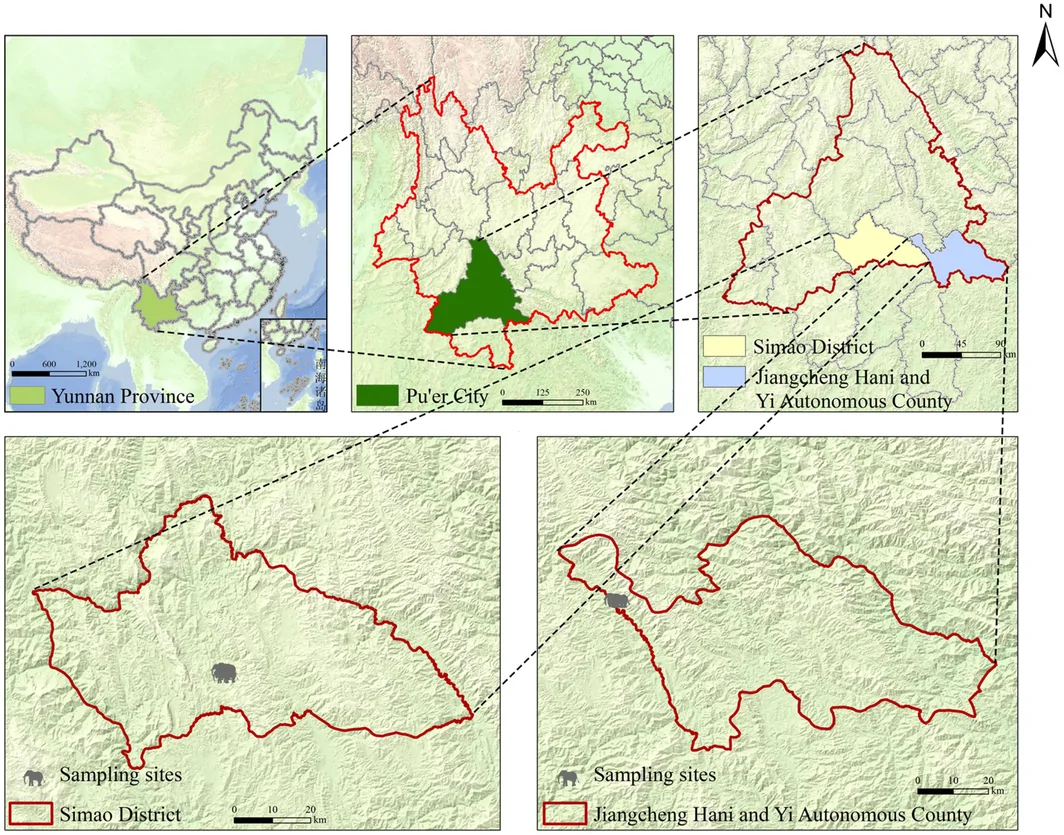
Research location map.

The Pu'er region in Yunnan Province is an important distribution area for Asian elephants in China (Figure 1), with different areas within this region exhibiting notable differences in vegetation composition, water source distribution, and intensity of human activity disturbance. Wild Asian elephants exhibit strict selectivity for habitat factors such as food type and quantity, habitat area, concealment conditions, water availability, and temperature, and they mainly inhabit tropical rainforests, seasonal rainforests, and forested ravines and thickets below an altitude of 1000 m (Tan and Yu 2015). The Jiangcheng subpopulation consists of approximately 44 elephants, while the Liushun subpopulation consists of approximately 45 elephants, and both interact with the Mengyang subpopulation in Mengwang Township, Jinghong City (Zhu et al. 2019). The Jiangcheng area is dominated by tropical seasonal evergreen broadleaf forest, which provides relatively abundant and stable food resources year‐round (Zhang et al. 2003); in contrast, the Liushun area experiences greater human disturbance, primarily in the form of agricultural expansion, crop cultivation, and increased infrastructure development, which lead to more pronounced spatial and seasonal fluctuations in food resource availability (Zhang et al. 2003; Guo et al. 2006). Currently, research on the gut microbiota of Asian elephants remains focused on wild and captive populations in the Xishuangbanna region, while studies on Asian elephants in the Pu'er region are relatively lacking.

## 3 Materials and Methods

### 3.1 Sample Collection

Fresh fecal samples of wild Asian elephants were collected from Jiangcheng Hani and Yi Autonomous County (JC, approximately 22.5° N, 101.8° E) and Liushun Town, Simao District (LS, approximately 22.7° N, 101.0° E), Pu'er City, Yunnan Province, between April and August 2025, with a total of 33 samples (21 from JC and 12 from LS). The collection was carried out by the Terrestrial Wildlife Epidemic Disease Surveillance Technical Support Team of the Yunnan Academy of Forestry and Grassland Sciences. Wild elephant monitors used the “Wild Asian Elephant Monitoring and Early Warning App” and drones to observe the herds and selected sites with frequent elephant activity and abundant food and water. The team waited for the elephants to enter and stay in the feeding areas, then drove to the sampling sites to observe and record the herds and collected fresh feces after the elephants defecated and left. To avoid repeated sampling from the same individual, the team coordinated with local monitors familiar with the herds, distinguished different individuals based on natural physical markers (such as ear tears, tusk shape, and body size) and the monitors' identification experience while also recording the spatial distribution and activity timing of the herds using drone flight logs. During the same sampling event, only fresh feces from different locations within the herd's ranging area were collected to minimize the probability of resampling the same elephant. All sampling was conducted in the early morning. Freshly excreted feces were selected, and approximately 5 g of the middle portion was collected using disposable sterile gloves, placed into 50 mL sterile centrifuge tubes, avoiding contact with the ground and surrounding environment to reduce contamination. All samples were immediately transported on dry ice after collection and stored in an ultra‐low temperature freezer at −80°C in the laboratory until further use. While the JC and LS subpopulations are known to interact with the Mengyang subpopulation, the Mengyang group was not included in this study due to logistical constraints and the specific research focus on contrasting the gut microbiota of elephants inhabiting areas with differing degrees of human disturbance and habitat heterogeneity as represented by the JC and LS sites.

### 3.2 DNA Extraction and PCR Amplification

Total DNA was extracted from fecal samples using the MOBIO PowerSoil DNA Kit following the manufacturer's instructions. The V3–V4 hypervariable region of the bacterial 16S rRNA gene was amplified using primers 341F and 806R (see Table 1). PCR products were examined by electrophoresis on 1% agarose gels, and qualified products were purified for library construction.

**TABLE 1:** PCR primer information.

Primer name	Sequence (5′ → 3′)
341F	ACTCCTACGGGAGGCAGCA
806R	CGTCAGACTTTCGTCCATTGC

### 3.3 Sequencing and Data Processing

PCR products were used to construct libraries with the NEBNext Ultra II DNA Library Prep Kit (New England Biolabs, USA). After the library quality was confirmed as qualified by Qubit quantification and Agilent 2100 analysis, paired‐end sequencing (PE250) was performed on the Illumina NovaSeq 6000 platform. The raw sequencing data were first subjected to quality control. Fastp software was used to filter sequences, remove adapters, and perform quality trimming to obtain high‐quality clean reads (Chen, Zhou, et al. 2018; Chen, Jiang, et al. 2018). Subsequently, the USEARCH software was employed for bidirectional sequence assembly and denoising processing, followed by OTU clustering based on a 97% similarity threshold (Edgar 2013).

### 3.4 Statistical Methods

The representative sequence of each OTU was aligned against the SILVA (16S) database using usearch‐sintax/blast to obtain taxonomic annotation information (confidence threshold default = 0.8, default database = SILVA (16S)) (Caporaso et al. 2010). Relative abundances of species were calculated at the phylum, class, order, family, genus, and other taxonomic levels, and community structure analyses were performed. Based on the OTU abundance table, alpha diversity indices (Chao1, Shannon, Simpson) were calculated using usearch‐alpha_div (V10, http://www.drive5.com/usearch/) and R packages to evaluate the species richness and diversity of the gut microbiota in the samples. Differences between groups were analyzed using the Wilcoxon rank‐sum test, with *p* < 0.05 considered statistically significant. Principal coordinate analysis (PCoA) was performed using the vegan package in R software with the Bray–Curtis distance algorithm to compare differences in microbial community structure between groups.

Based on the normalized abundance table (OTU_table) at various taxonomic levels, LEfSe (Linear Discriminant Analysis Effect Size) software was used to identify differentially abundant species between groups. First, the nonparametric factorial Kruskal–Wallis (KW) sum‐rank test was used to detect species with significant abundance differences among groups. Then, the pairwise Wilcoxon rank‐sum test was applied to determine differences between two groups. Finally, linear discriminant analysis (LDA) was used for dimensionality reduction and to evaluate the effect size of differentially abundant species (i.e., LDA score). The default LDA score threshold was set to 2, and the results represent the biomarkers within each group. The OTU abundance table was normalized using PICRUSt2 to remove the effect of copy number of the 16S marker gene in species genomes. Then, through the GreenGene ID corresponding to each OTU/ASV, the data were aligned to the KEGG database. Based on the KEGG annotation, KO, Pathway, and EC information were obtained, and the abundance of each functional category was calculated according to OTU abundance. Abundance tables for each sample at different functional levels were generated for subsequent analyses (Langille et al. 2013).

## 4 Results and Analysis

### 4.1 Analysis of Species Community Structure

**FIGURE 2:**
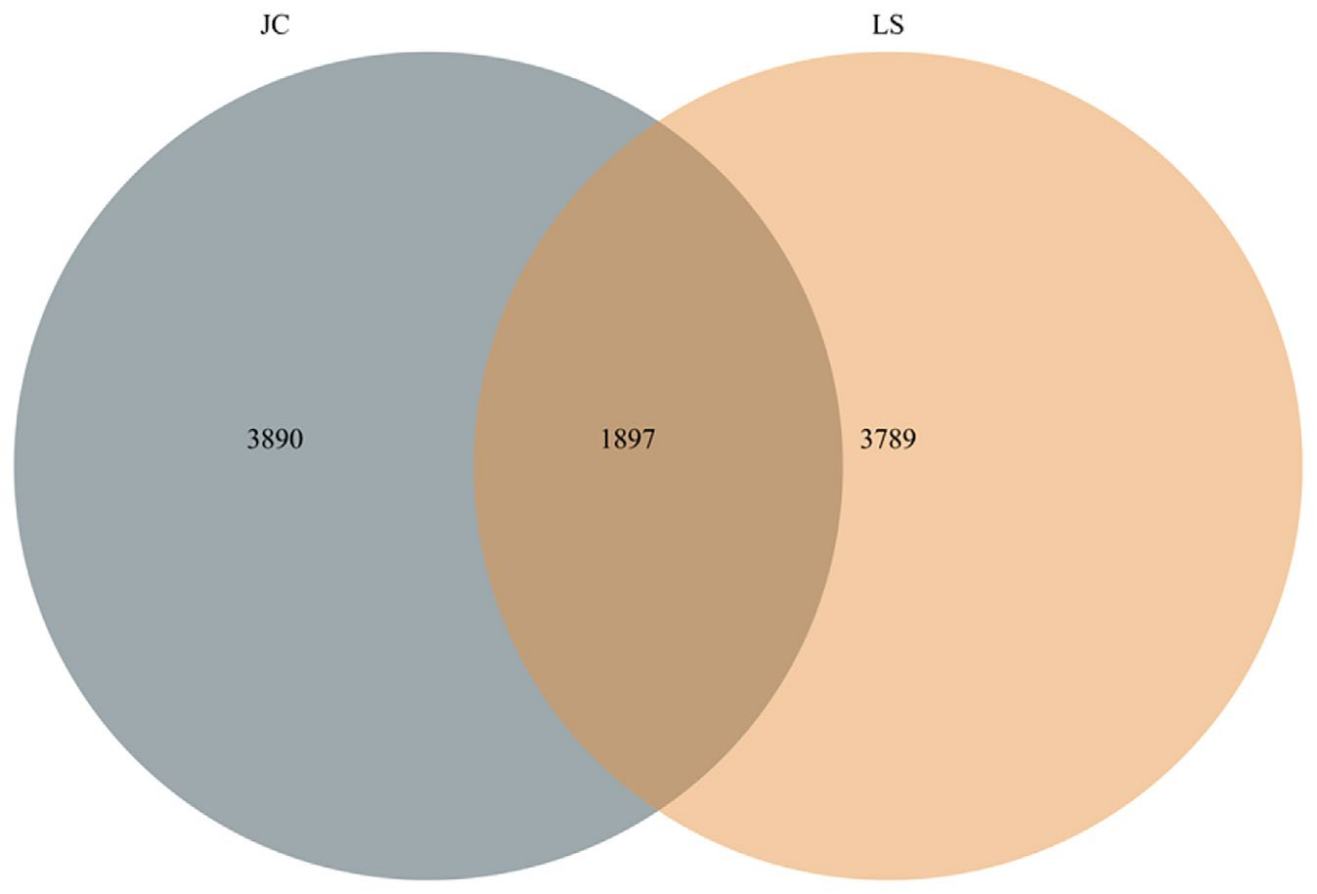
Venn diagram of shared and unique OTUs.

**FIGURE 3:**
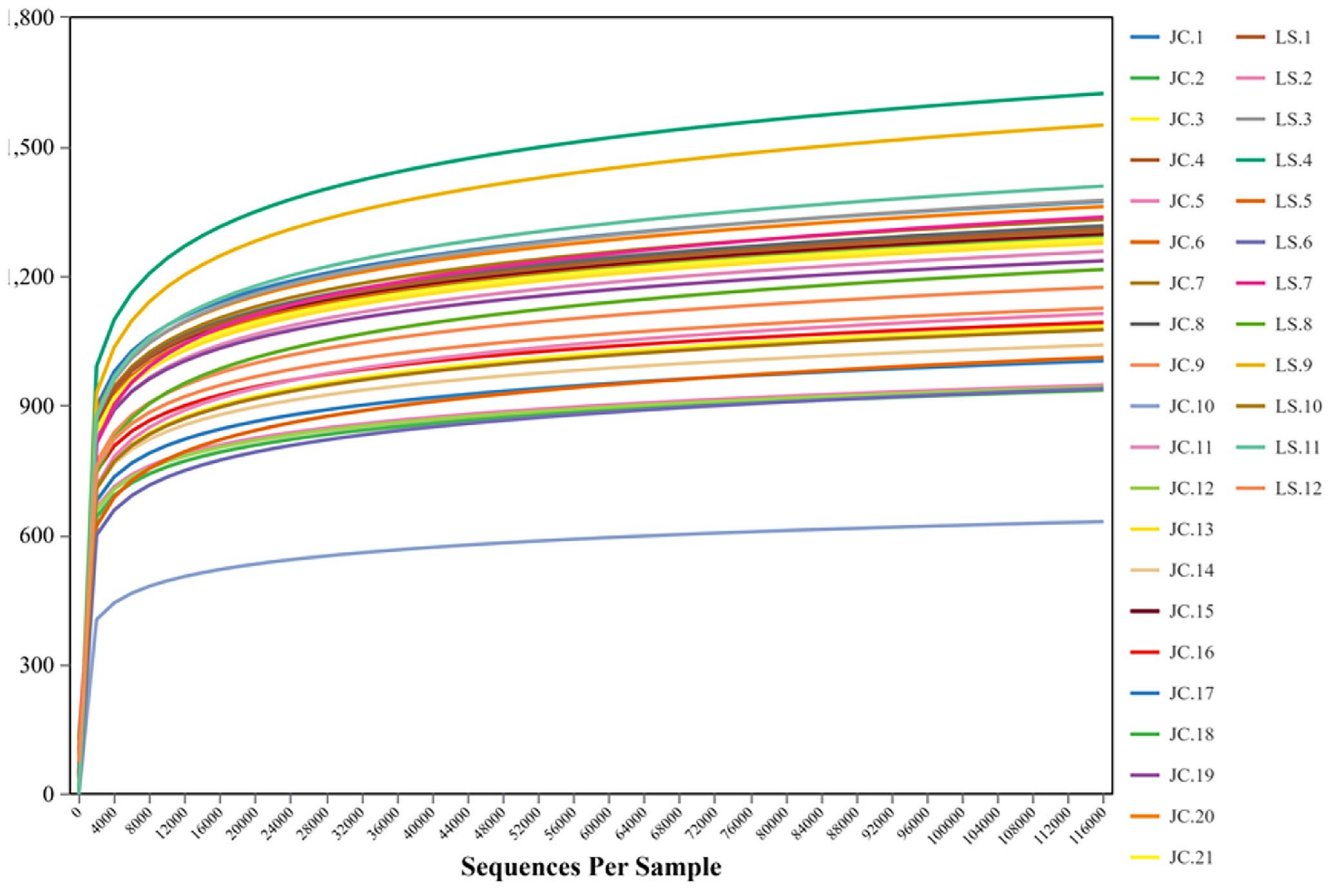
Rarefaction curves.

Based on a 97% similarity threshold, OTU clustering analysis was performed on a total of 3,334,093 sequences from the JC and LS groups using the UPARSE algorithm implemented in USEARCH v10 (Edgar 2013) to obtain the community composition at various taxonomic levels. During clustering, sequences with lengths < 400 or > 600 bp were discarded, and the maximum mismatches in the barcode were set to 1. Among all OTUs obtained from the samples, a total of 24 phyla, 48 classes, 98 orders, 164 families, 288 genera, and 351 species were annotated. The number of shared OTUs between the two groups was 1897; the JC group had 3890 unique OTUs, while the LS group had 3789 unique OTUs (Figure 2), indicating significant differences in the species composition of the gut microbiota of Asian elephants under different habitat conditions. The representative sequence of each OTU was aligned against the SILVA v138 (16S) database using usearch‐sintax/blast with a default confidence threshold of 0.8 to obtain taxonomic annotation information. The rarefaction curves (Figure 3) showed that as sequencing depth increased, the curves for each sample gradually leveled off and eventually reached a plateau, indicating that the sequencing data volume in this study had largely covered the vast majority of microbial species in the samples and that the sequencing depth was sufficient to truly reflect the diversity characteristics of the microbial communities.

### 4.2 Analysis of Species Community Composition

While genus‐level taxonomy is commonly emphasized in microbiome studies, we present our compositional data at the phylum and family levels in the main text. This choice is primarily motivated by our LEfSe analysis, which identified specific families (e.g., Flavobacteriaceae and Bacteroidaceae) as the strongest discriminant taxa between the JC and LS groups (Figure S1). Focusing on these levels allows a more direct integration with the key biomarkers and facilitates the ecological interpretation of habitat‐driven differences.

**FIGURE 4:**
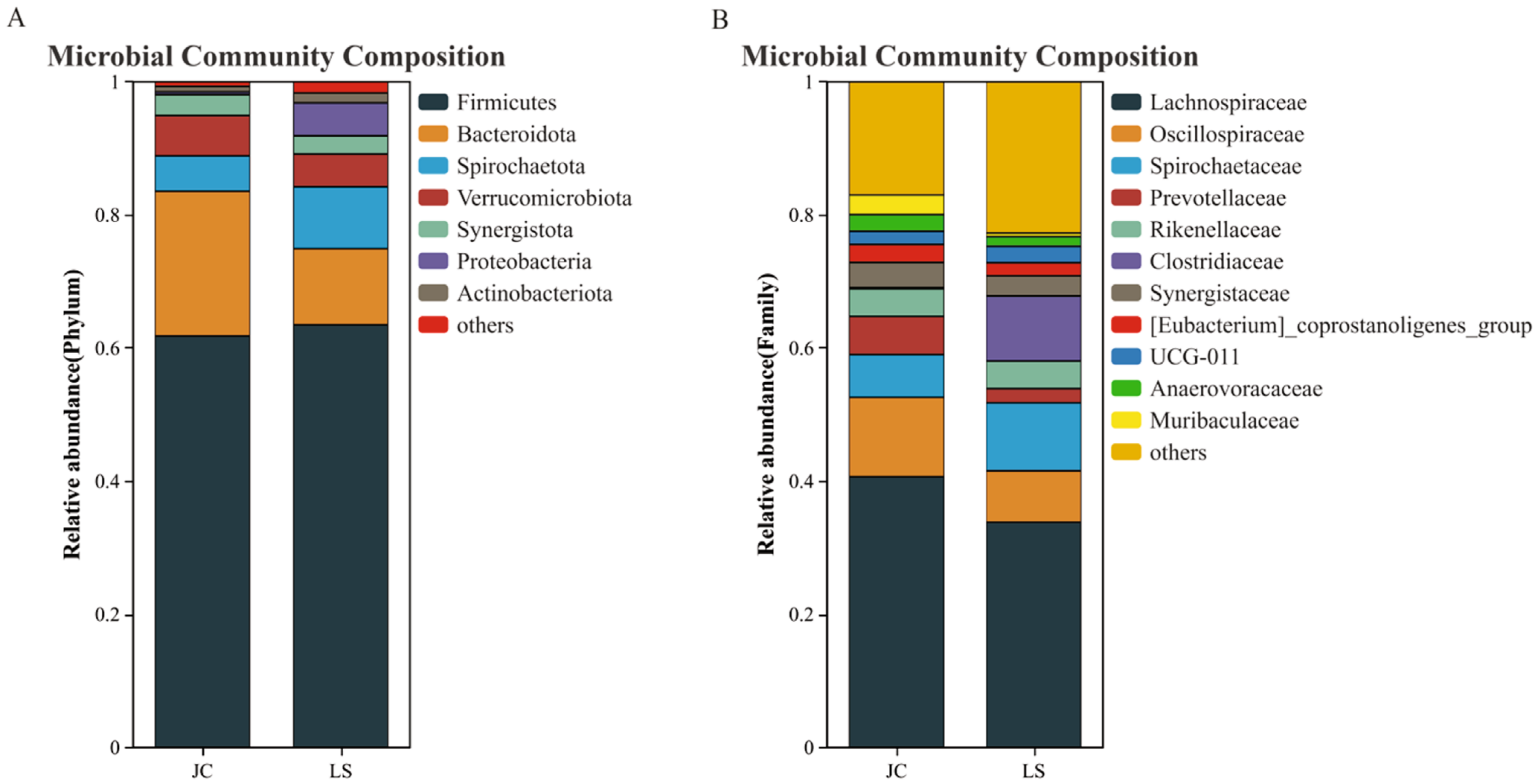
Composition of the gut microbiota in wild Asian elephants. (A) Phylum level. (B) Family level. Data are presented as mean ± SD for each group at the phylum and family levels.

At the phylum level (Figure 4A), the top four dominant phyla in the JC group were Firmicutes (62.05% ± 9.46%), Bacteroidota (21.60% ± 10.25%), Verrucomicrobiota (5.93% ± 4.23%), and Spirochaetota (5.30% ± 2.50%). In the LS group, the top four dominant phyla were Firmicutes (63.52% ± 6.73%), Bacteroidota (11.29% ± 4.17%), Proteobacteria (5.40% ± 9.58%), and Verrucomicrobiota (4.98% ± 3.72%). Notably, Proteobacteria was a uniquely dominant phylum in the LS group, albeit with high interindividual variation (SD > mean).

At the family level (Figure 4B), the top four dominant families in the JC group were Lachnospiraceae (40.42% ± 5.80%), Oscillospiraceae (12.12% ± 4.56%), Spirochaetaceae (6.42% ± 2.82%), and Prevotellaceae (5.66% ± 4.56%). In the LS group, the top four dominant families were Lachnospiraceae (33.38% ± 11.85%), Spirochaetaceae (9.62% ± 7.44%), Clostridiaceae (9.58% ± 13.06%), and Oscillospiraceae (8.34% ± 5.27%). Among these, Clostridiaceae was a uniquely dominant family in the LS group, though its high standard deviation indicates substantial interindividual variation.

### 4.3 Alpha Diversity Analysis

**FIGURE 5:**
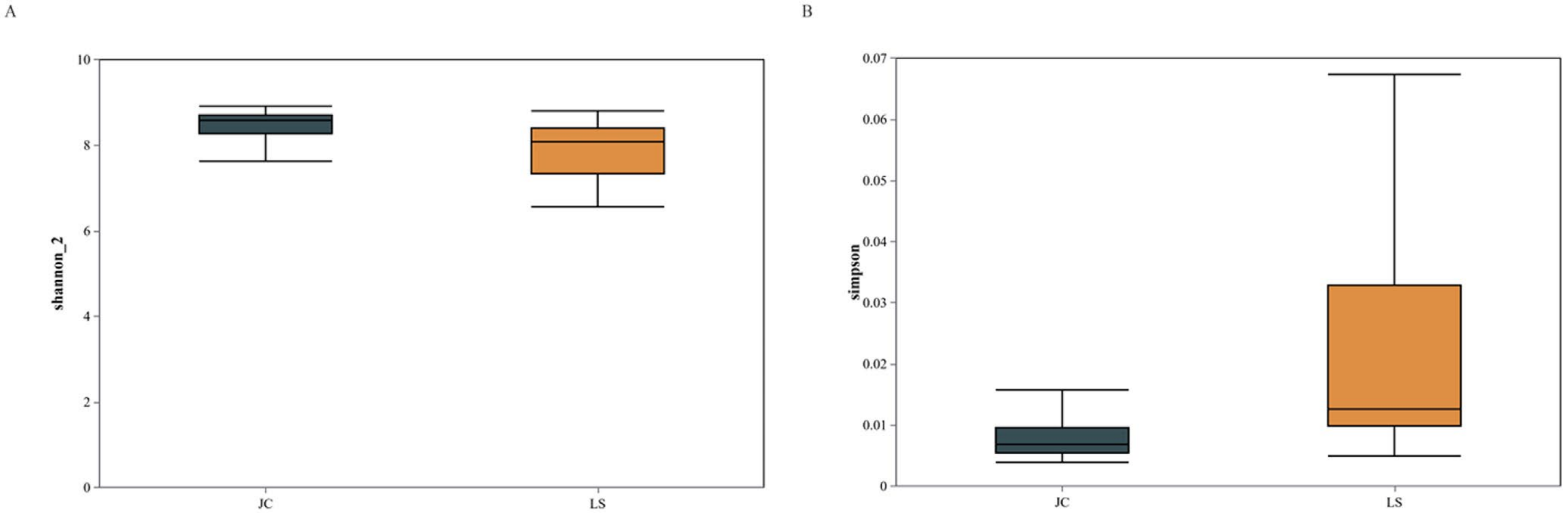
Boxplot of intergroup difference analysis. (A) Shannon index. (B) Simpson index.

The results of alpha diversity analysis showed that there was no significant difference in gut microbiota richness between the two groups of wild Asian elephants, but significant differences were observed in community diversity (Table 2). Specifically, both the Shannon index (*p* = 0.028) and the Simpson index (*p* = 0.013) reached significant levels between the two groups (*p* < 0.05), indicating that the gut microbiota diversity of the JC group was significantly higher than that of the LS group. As shown in Figure 5, the Shannon index values of the JC group were relatively concentrated with low dispersion, suggesting a relatively stable gut microbiota structure. In contrast, the LS group exhibited a wider range and higher dispersion of index values, indicating greater within‐group variation. The LS group showed overall higher and more fluctuating Simpson index values, implying a higher proportion of dominant taxa in the community, whereas the JC group displayed lower and more stable Simpson index values, reflecting a more even species distribution.

**TABLE 2:** Results of alpha diversity index difference test.

Index	*p*
Chao1	0.238
Shannon	0.028
Simpson	0.013

### 4.4 Beta Diversity Analysis

**FIGURE 6:**
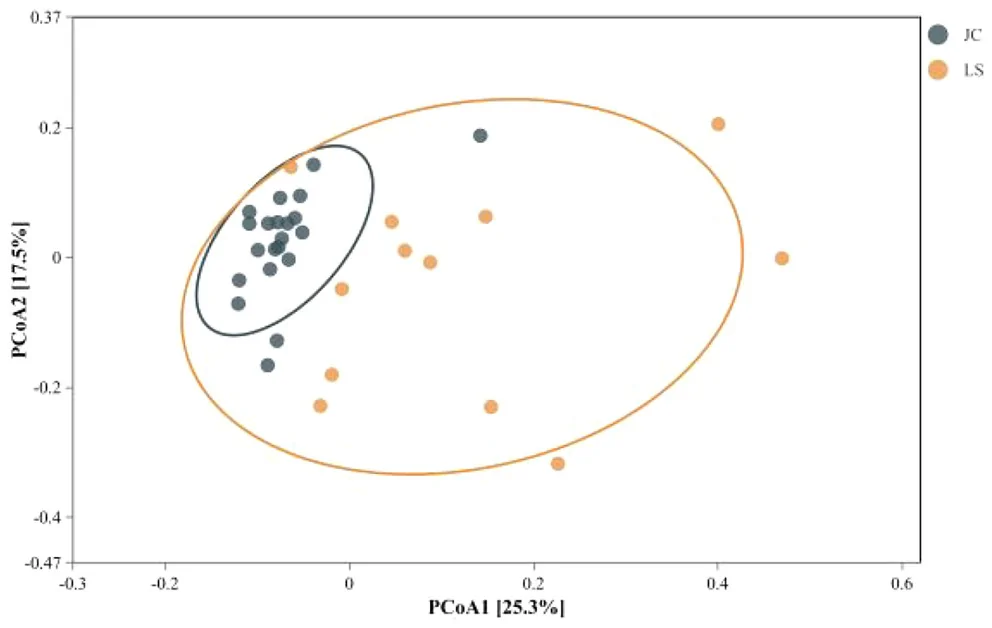
PCoA plot.

Principal coordinate analysis (PCoA) based on Bray–Curtis distance (Figure 6) showed that the JC group had a relatively concentrated distribution with a smaller confidence ellipse, indicating relatively homogeneous environmental conditions at this site, high stability of community structure, and high similarity in species composition among samples. In contrast, the LS group exhibited a more dispersed distribution with a larger confidence ellipse, suggesting higher environmental heterogeneity at this site and significant spatial variation in community structure. Simultaneously, the ANOSIM test at the genus level revealed highly significant differences in gut microbiota composition between the two groups (Table 3; ANOSIM, *R* = 0.421, *p* = 0.001 < 0.01).

**TABLE 3:** Comparison of gut microbiota diversity between two groups based on ANOSIM test.

Comparison groups	*R*	*p*
JC vs. LS	0.421	0.001

### 4.5 LEfSe Analysis

Red represents the JC group and green represents the LS group. In the JC group, Flavobacteriaceae (LDA≈4.2) contributed the most to the differences, while in the LS group, Bacteroidaceae (LDA≈4.0) contributed the most. The number of differential species in the JC group was greater than that in the LS group, indicating that the JC group harbored a richer set of characteristic microbial taxa (see Figure S1 for the LDA score distribution bar plot).

### 4.6 KEGG Functional Prediction Analysis

Based on functional prediction of gut microbial communities using PICRUSt2 and alignment with the KEGG database, this study compared the functional profile differences between the JC and LS groups at both Level 1 and 2, while employing Wilcoxon rank‐sum tests to assess the significance of intergroup differences for each functional pathway.

**FIGURE 7:**
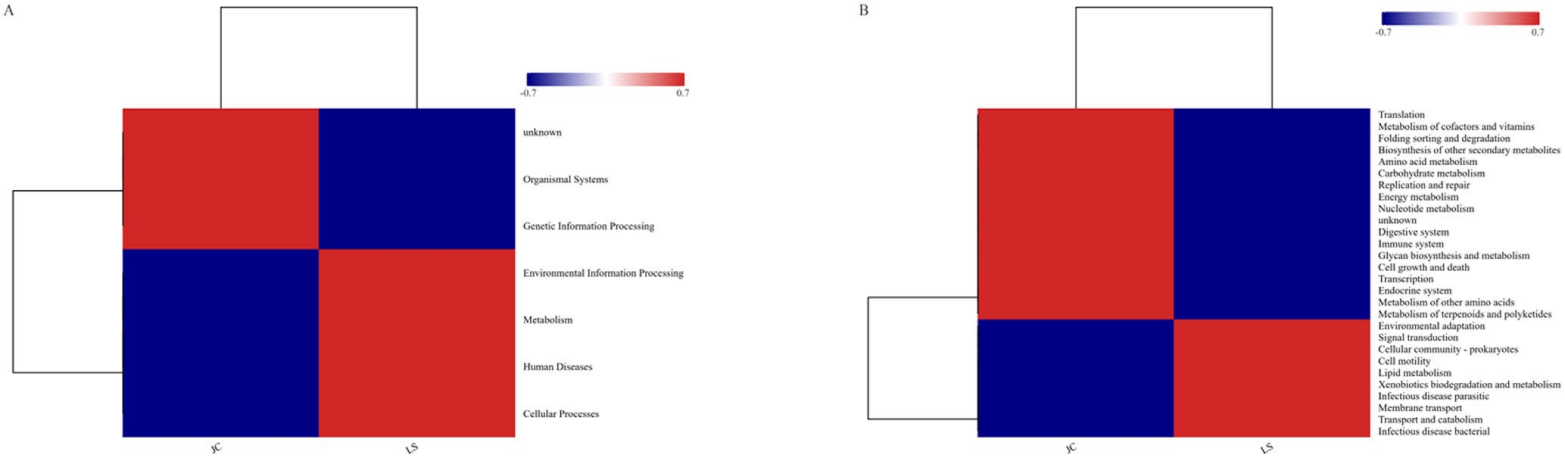
Heatmap of KEGG abundance clustering. (A) Level 1. (B) Level 2.

At KEGG Level 1 (Figure 7A; Table 4), both groups exhibited “Metabolism” as the predominant functional category (JC: 0.7668 ± 0.0070; LS: 0.7707 ± 0.0082), followed by “Genetic Information Processing” (JC: 0.1458 ± 0.0039; LS: 0.1386 ± 0.0040) and “Cellular Processes” (JC: 0.0542 ± 0.0053; LS: 0.0559 ± 0.0045). The JC group showed significantly higher abundance in “Genetic Information Processing” (*p* = 0.031), whereas the LS group showed significantly higher abundance in “Environmental Information Processing” (*p* = 0.027). These results indicate clear intergroup functional differentiation at Level 1.

**TABLE 4:** Relative abundance (mean values) of KEGG Level 1 functional pathways and Wilcoxon rank‐sum test results.

Level 1	JC (Mean ± SD)	LS (Mean ± SD)	*p*
Metabolism	0.7668 ± 0.0070	0.7707 ± 0.0082	0.684
Genetic information processing	0.1458 ± 0.0039	0.1386 ± 0.0040	0.031
Cellular processes	0.0542 ± 0.0053	0.0559 ± 0.0045	0.472
Environmental information processing	0.0242 ± 0.0016	0.0261 ± 0.0019	0.027

*Note:* Data are presented as mean ± standard deviation (SD) for each group (JC, *n* = 21; LS, *n* = 12). Significant differences (*p* < 0.05) are highlighted in bold based on Wilcoxon rank‐sum tests.

At KEGG Level 2 (Figure 7B; Table 5), a total of 15 functional subcategories were annotated across both groups. The JC group exhibited higher abundance in pathways including “Carbohydrate metabolism” (0.1472 ± 0.0023 vs. 0.1435 ± 0.0053), “Amino acid metabolism” (0.1326 ± 0.0031 vs. 0.1293 ± 0.0030), “Metabolism of cofactors and vitamins” (0.1256 ± 0.0059 vs. 0.1188 ± 0.0041), “Energy metabolism” (0.0521 ± 0.0018 vs. 0.0508 ± 0.0027), and “Replication and repair” (0.0647 ± 0.0021 vs. 0.0610 ± 0.0024), collectively reflecting a functional bias toward genetic information transfer, energy generation, and basic biosynthesis. In contrast, the LS group showed higher abundance in pathways such as “Lipid metabolism” (0.0590 ± 0.0082 vs. 0.0504 ± 0.0020), “Cell motility” (0.0366 ± 0.0043 vs. 0.0340 ± 0.0054), and “Membrane transport” (0.0214 ± 0.0018 vs. 0.0200 ± 0.0014), as well as pathways related to “Environmental adaptation,” “Signal transduction,” and “Xenobiotics biodegradation,” reflecting stronger environmental adaptation and stress response characteristics. Wilcoxon rank‐sum tests indicated that “Energy metabolism” (*p* = 0.042), “Signal transduction” (*p* = 0.038), and “Xenobiotics biodegradation and metabolism” (*p* = 0.029) differed significantly between the two groups. Although other functional pathways exhibited intergroup differences in abundance, these did not reach statistical significance (*p* > 0.05).

**TABLE 5:** Relative abundance (mean values) of KEGG Level 2 functional pathways and Wilcoxon rank‐sum test results (only the top 15 subcategories are listed).

Level 2	JC (Mean ± SD)	LS (Mean ± SD)	*p*
Carbohydrate metabolism	0.1472 ± 0.0023	0.1435 ± 0.0053	0.218
Amino acid metabolism	0.1326 ± 0.0031	0.1293 ± 0.0030	0.305
Metabolism of cofactors and vitamins	0.1256 ± 0.0059	0.1188 ± 0.0041	0.194
Metabolism of terpenoids and polyketides	0.1047 ± 0.0020	0.1036 ± 0.0076	0.876
Metabolism of other amino acids	0.0657 ± 0.0026	0.0644 ± 0.0023	0.438
Replication and repair	0.0647 ± 0.0021	0.0610 ± 0.0024	0.151
Lipid metabolism	0.0504 ± 0.0020	0.0590 ± 0.0082	0.087
Energy metabolism	0.0521 ± 0.0018	0.0508 ± 0.0027	0.042
Translation	0.0364 ± 0.0010	0.0341 ± 0.0014	0.367
Cell motility	0.0340 ± 0.0054	0.0366 ± 0.0043	0.196
Glycan biosynthesis and metabolism	0.0352 ± 0.0034	0.0305 ± 0.0017	0.214
Folding, sorting and degradation	0.0323 ± 0.0010	0.0319 ± 0.0006	0.672
Biosynthesis of other secondary metabolites	0.0212 ± 0.0016	0.0208 ± 0.0021	0.751
Nucleotide metabolism	0.0216 ± 0.0006	0.0204 ± 0.0006	0.408
Membrane transport	0.0200 ± 0.0014	0.0214 ± 0.0018	0.104

*Note:* Data are presented as mean ± standard deviation (SD) for each group (JC, *n* = 21; LS, *n* = 12). Significant differences (*p* < 0.05) are highlighted in bold based on Wilcoxon rank‐sum tests.

Overall, the gut microbiota of the JC group was functionally biased toward genetic information processing and energy metabolism, suggesting that its microbial community possesses high metabolic activity and functional stability. In contrast, the LS group was enriched in pathways related to environmental information processing, signal transduction, and xenobiotic degradation, reflecting a stronger adaptive response of its gut microbiota to environmental fluctuations and anthropogenic disturbance pressures. This functional differentiation is consistent with the differences in habitat conditions (food resource stability, intensity of human disturbance) between the two groups, further supporting the shaping effect of habitat heterogeneity on the gut microbial ecological functions of Asian elephants.

## 5 Discussion

The wild Asian elephant is a first‐class nationally protected wildlife species in China. Its gut microbiota structure is closely linked to host nutritional metabolism, immune regulation, and environmental adaptability. The Pu'er region is an important distribution area for Asian elephants, where different subpopulations face varying habitat conditions (e.g., vegetation composition, intensity of anthropogenic disturbance). However, systematic research on the gut microecology of Asian elephants in this region remains scarce. This study compared the fecal microbial composition, diversity, and function of wild Asian elephants from Jiangcheng (JC) and Liushun (LS), aiming to reveal the adaptive response mechanisms of the gut microbiota to heterogeneous habitats, assess their relationship with host health and habitat quality, and provide a microecological basis for conservation management, habitat restoration, and health assessment of Asian elephants.

At the phylum level, Firmicutes and Bacteroidetes were the common dominant phyla in both groups, a finding consistent with previous studies (Zhang et al. 2019; Keady et al. 2021). In African savanna elephants and African forest elephants, the most prevalent phyla were Firmicutes, Proteobacteria, and Bacteroidetes; the gut microbiota of savanna elephants was dominated by Firmicutes, whereas that of forest elephants was dominated by Proteobacteria, which is associated with dietary differences driven by their distinct habitats (Budd et al. 2020). Firmicutes and Bacteroidetes are major components of the gut microbiota in these animals and are essential for fermentation of plant‐derived polysaccharides (Budd et al. 2020; Berg and Cernava 2022; Niu et al. 2023). Proteobacteria were uniquely detected in the LS group but not in the JC group. It has been reported that Proteobacteria are involved in protein and lipid metabolism in the intestines of ruminants and poultry (Cholewińska et al. 2020), suggesting that Asian elephants in the LS group may consume more energy. Notably, Proteobacteria are known to be enriched under conditions of host stress, dietary imbalance, or low‐grade intestinal inflammation (Shin et al. 2015). In the LS area, where human disturbance is stronger and food resources fluctuate more, the enrichment of Proteobacteria may reflect a state of gut microbial dysbiosis or an adaptive shift in the microbiota to cope with fluctuating nutrient availability and environmental stressors. This observation is consistent with studies in other mammals, where Proteobacteria abundance often increases in response to dietary changes or anthropogenic pressures (Zhu et al. 2022). Furthermore, the abundance of Spirochaetes was lower in the LS group than in the JC group. Previous studies have shown that under high‐fiber dietary conditions, Spirochaetes can cooperate with Bacteroidetes to promote carbohydrate metabolism, but their abundance may become abnormal under specific disease conditions or environmental stress (Shoaib et al. 2024). Therefore, monitoring changes in gut microbial abundance in wild Asian elephants is of great value for health assessment.

At the family level, both groups shared Lachnospiraceae, Spirochaetaceae, and Oscillospiraceae as dominant families, together accounting for over 50% of the gut microbiota and constituting the major components. This result is consistent with a previous study (Yang 2025). A characterization study of gut microbiota in wild Asian elephants from five nature reserves in Thailand similarly found that Lachnospiraceae was the most dominant family across all sampling sites, followed by Oscillospiraceae (Kantirakoon et al. 2026). As a key fiber‐degrading group within Firmicutes, Lachnospiraceae is widespread in the intestines of large herbivores and plays a critical role in the fermentation of plant cell wall polysaccharides (Wang, Wang, Zhou, et al. 2024). Regarding Oscillospiraceae, a study reported high abundance of Oscillospira in the gut of plateau pikas, which is associated with plant polysaccharide degradation (Feng et al. 2020), implying that Oscillospira may participate in the fermentation and metabolism of dietary fiber. Spirochaetaceae, due to their unique spiral morphology and active motility, are widely distributed in natural environments and within animal bodies (Pappas et al. 2024). Overall, the abundant presence of Lachnospiraceae, Oscillospiraceae, and Spirochaetaceae in the gut of wild Asian elephants is crucial for health maintenance and energy acquisition, although potential pathogenic risks also warrant attention. In this study, the abundance of Prevotellaceae was significantly higher in the JC group than in the LS group. Prevotella is a key taxon in the herbivore gut that degrades complex carbohydrates and can utilize dietary fiber, resistant starch, and other polysaccharides to produce short‐chain fatty acids (e.g., acetate, propionate, and butyrate), which positively affect host energy metabolism and intestinal health (Ley 2016). In a dietary study on juvenile Asian elephants, Prevotellaceae was more abundant in the goat milk–plant mixed‐feed group, further confirming the association of this family with plant‐based diet intake (Zhang et al. 2023). Based on these findings, the higher abundance of Prevotellaceae in the JC group may be related to the relatively stable and sufficient high‐fiber food resources available in their habitat, which could positively influence their intestinal metabolism and health. In addition to dietary factors, gut microbial composition is also closely associated with host developmental stage. A study on Asian elephants at Okinawa Zoo in Japan found that the gut microbial diversity of calves was significantly lower than that of their mothers and gradually increased with age (Kambe et al. 2020). A study on captive Asian elephants in Germany also showed that gut microbial richness was significantly lower in suckling calves than in plant‐eating individuals (Imlberger et al. 2014). In the wild, calves supplement their gut microbiota by consuming feces from adult elephants (Chen, Zhou, et al. 2018; Chen, Jiang, et al. 2018), a behavior that is important for the establishment and development of the gut microbial community in juveniles.

Significant differences in gut microbial diversity were observed between the two groups. The alpha diversity of the JC group was significantly higher than that of the LS group. Beta diversity analysis further revealed that samples from the JC group were more clustered, indicating higher community stability, whereas samples from the LS group were more dispersed, indicating greater spatial variation. Previous studies have shown that vegetation type, food sources, and environmental disturbance are important factors driving differences in gut microbial structure (Budd et al. 2020; Rana et al. 2020). In a comparative study of North American bison ( *Bison bison* ) subspecies (plains bison and wood bison), subspecies classification had no significant effect on alpha diversity but significantly affected beta diversity, indicating that gut microbial differences are primarily driven by differential abundance of specific taxa, likely influenced by dietary preferences, ancestral phenotypes, and historical range distributions. This pattern echoes the beta‐diversity differences observed between the JC and LS groups in our study (Grant et al. 2025). Another study based on 16S rRNA gene phylogenetic analysis found that while alpha diversity of gut bacterial communities in wild Asian elephants showed no significant difference between rainy and dry seasons (remaining generally stable), beta diversity differed significantly, suggesting that seasonal changes (e.g., food availability, temperature, humidity) influence species composition (Chen 2025). Since all samples in the present study were collected during the early to mid‐rainy season and dry‐season samples were not included, this limits a comprehensive understanding of seasonal dynamics in the gut microbiota. To address this limitation, future studies should include dry‐season samples to complete the seasonal sequence and enable systematic comparison of microbial composition and function across seasons.

LEfSe analysis revealed that Flavobacteriaceae contributed most to the differences in the JC group. In herbivorous wildlife, Flavobacteriaceae are commonly associated with plant fiber degradation and breakdown of complex polysaccharides (Lee et al. 2024). Their higher contribution in the JC group likely reflects the relatively stable and abundant high‐fiber food resources available in the intact tropical forests of the JC area, which may favor the proliferation of this fiber‐degrading family. In contrast, Bacteroidaceae were the most differentially abundant family in the LS group. Bacteroidaceae are core functional groups in the gut that efficiently degrade dietary fiber and mucosal glycans, playing key roles in maintaining the intestinal barrier and regulating host immunity (Wexler 2007; Flint et al. 2012). The prominent contribution of Bacteroidaceae in the LS group may indicate a greater need for efficient energy extraction from a more variable diet, or it may reflect an adaptive response of the host to nutritional stress and environmental fluctuations caused by anthropogenic disturbance.

Functional prediction analysis revealed a clear divergence between the two groups at the KEGG pathway level. The JC group was mainly enriched in pathways related to genetic information processing and energy metabolism, reflecting higher metabolic activity and functional stability of their gut microbiota. In contrast, the LS group was enriched in pathways associated with environmental information processing, signal transduction, and xenobiotic metabolism, suggesting that their gut microbiota is more oriented toward functions related to environmental adaptation. Previous studies have shown that gut microbial communities can undergo functional reorganization in response to changes in host diet and external environmental stress (Imlberger et al. 2014; Qin 2007), which is consistent with the findings of this study. In a metagenomic survey of the fecal microbiome of African savanna elephants, carbohydrate catabolic pathways were identified as the main degradation pathways, and the fecal metagenome was enriched in glycoside hydrolase (GH) class CAZymes, with GH43, GH2, GH13, and GH3 being the most abundant GH families (du Preez et al. 2024). Future research could adopt this metagenomic approach to further dissect the actual functional differences between the two groups and their ecological significance.

This study has several limitations in individual identification, taxonomic resolution, and study design. To address these, future research should employ fecal DNA microsatellite or genomic marker‐based individual identification to minimize resampling risks. Metagenomics and metatranscriptomics should be adopted to overcome the taxonomic and functional inference constraints of 16S rRNA sequencing, enabling direct characterization of functional gene profiles and metabolic activities. Longitudinal time‐series sampling covering complete dry and rainy seasons, combined with fixed‐site continuous monitoring, is needed to reveal seasonal succession patterns and microbial responses to environmental fluctuations. Additionally, integrating fecal microhistology, stable isotope analysis, and environmental DNA metabarcoding will allow precise quantification of dietary composition and establishment of multivariate association models linking environmental parameters (e.g., food resource availability, vegetation type, anthropogenic disturbance intensity) to microbial features, thereby providing direct causal evidence for habitat heterogeneity driving gut microbiota differences. These efforts will deepen our understanding of the ecological adaptive mechanisms of gut microbiota in wild Asian elephants and offer a more robust microecological foundation for habitat quality assessment, health monitoring, and conservation management.

## 6 Conclusion

In summary, this study provides empirical evidence that habitat heterogeneity significantly influences the gut microbiota of wild Asian elephants. Specifically, elephants in the JC area, inhabiting relatively intact forests with stable food resources, exhibited higher gut microbial diversity, more stable community structure, and enhanced fiber‐degrading and energy metabolism capabilities. In contrast, the LS group, subjected to greater anthropogenic disturbance and fluctuating food availability, displayed a microbiota oriented toward environmental adaptation and stress responses. These findings highlight the potential use of gut microbiota profiling as a sensitive bioindicator for habitat quality assessment in Asian elephants. From a conservation perspective, maintaining habitat integrity and ensuring stable food resources are critical not only for the nutritional health of elephant populations but also for the preservation of a healthy and functionally diverse gut microecosystem. This study thus provides a microecological foundation for evidence‐based habitat management and ex situ conservation strategies for this endangered species.

## Author Contributions

**Zheng Jing:** conceptualization (lead), formal analysis (equal), investigation (equal), visualization (lead), writing – original draft (lead). **Zhou Jiuxuan:** funding acquisition (equal), project administration (equal), resources (equal). **Liu Xia:** project administration (equal), supervision (equal), writing – review and editing (equal). **Wang Binghui:** investigation (equal), writing – review and editing (equal). **Wang Yixuan:** supervision (equal), writing – review and editing (equal). **Cao Hui:** investigation (equal), writing – review and editing (equal). **Lu Tihuo:** investigation (equal).

## Funding

This study was supported by Epidemic Source and Disease Detection and Early Warning for Terrestrial Wild Animals in Yunnan Province (Forestry and Grassland Disaster Prevention and Mitigation)‐2026 and the National Natural Science Foundation of China (no. 31860166).

## Ethics Statement

The animal studies were approved by the Pu'er City Forestry and Grassland Bureau and the Terrestrial Wildlife Epidemic Source and Disease Surveillance Technical Support Team of the Yunnan Academy of Forestry and Grassland Sciences, which granted approval for the participation of their animals in this study. The studies were conducted in accordance with local legislation and institutional requirements.

## Conflicts of Interest

The authors declare no conflicts of interest.

## Supporting information


**Figure S1:** Bar plot of LDA score distribution.

## Data Availability

All data generated or analyzed during this study are included in the NCBI BioProject database under accession number PRJNA1456346. The dataset can be accessed at https://www.ncbi.nlm.nih.gov/bioproject/PRJNA1456346.

## References

[ece374333-bib-0049] Berg, G. , and T. Cernava . 2022. “The Plant Microbiota Signature of the Anthropocene as a Challenge for Microbiome Research.” Microbiome 10, no. 1: 54–54. 35346369 10.1186/s40168-021-01224-5 PMC8959079

[ece374333-bib-0001] Budd, K. , J. C. Gunn , T. Finch , et al. 2020. “Effects of Diet, Habitat, and Phylogeny on the Fecal Microbiome of Wild African Savanna ( *Loxodonta africana* ) and Forest Elephants ( *L. cyclotis* ).” Ecology and Evolution 10, no. 12: 5637–5650. 32607180 10.1002/ece3.6305 PMC7319146

[ece374333-bib-0002] Caporaso, J. G. , J. Kuczynski , J. Stombaugh , et al. 2010. “QIIME Allows Analysis of High‐Throughput Community Sequencing Data.” Nature Methods 7, no. 5: 335–336. 20383131 10.1038/nmeth.f.303 PMC3156573

[ece374333-bib-0003] Chen, J. M. 2025. “Seasonal Variation of Gut Microbial Community and Function in Wild Asian Elephants.” Kunming: Yunnan Normal University.

[ece374333-bib-0004] Chen, M. Y. , Z. C. Jiang , F. Wang , et al. 2018. Asian Elephant Behavior Research. Yunnan Science and Technology Press.

[ece374333-bib-0005] Chen, S. , Y. Zhou , Y. Chen , et al. 2018. “Fastp: An Ultra‐Fast All‐In‐One FASTQ Preprocessor.” Bioinformatics 34, no. 17: i884–i890. 30423086 10.1093/bioinformatics/bty560 PMC6129281

[ece374333-bib-0007] Cholewińska, P. , K. Czyż , P. Nowakowski , et al. 2020. “The Microbiome of the Digestive System of Ruminants—A Review.” Animal Health Research Reviews 21, no. 1: 1–12. 31918781 10.1017/S1466252319000069

[ece374333-bib-0008] Chong, R. , C. E. Grueber , S. Fox , et al. 2019. “Looking Like the Locals: Gut Microbiome Changes Post‐Release in an Endangered Species.” Animal Microbiome 1, no. 1: 8. 33499935 10.1186/s42523-019-0012-4 PMC7807427

[ece374333-bib-0009] Edgar, R. C. 2013. “UPARSE: Highly Accurate OTU Sequences From Microbial Amplicon Reads.” Nature Methods 10, no. 10: 996–998. 23955772 10.1038/nmeth.2604

[ece374333-bib-0010] Feng, T. S. , Q. L. Yu , R. Zhou , et al. 2020. “Comparison of Gut Microbiome and Common Antibiotic Resistance Genes in Plateau Pika and Plateau Zokor.” Journal of Wildlife 41, no. 4: 897–911.

[ece374333-bib-0011] Flint, H. J. , K. P. Scott , S. H. Duncan , P. Louis , and E. Forano . 2012. “Microbial Degradation of Complex Carbohydrates in the Gut.” Gut Microbes 3, no. 4: 289–306. 22572875 10.4161/gmic.19897 PMC3463488

[ece374333-bib-0013] Grant, L. M. , M. R. Petri , M. T. Baecklund , et al. 2025. “Subspecific Variation in Gut Microbiota of North American Bison in a Sympatric Setting Reveals Differentially Abundant Taxa.” Animal Microbiome 7, no. 1: 89. 40841983 10.1186/s42523-025-00451-7 PMC12372302

[ece374333-bib-0014] Guo, Y. L. , L. Zhang , and Y. H. Dong . 2006. “Foraging Behavior of Wild Asian Elephants in Xishuangbanna.” Acta Theriologica Sinica 26, no. 1: 54–58.

[ece374333-bib-0015] Imlberger, N. , S. Güllert , J. Dannenberg , et al. 2014. “A Comparative Metagenome Survey of the Fecal Microbiota of a Breast‐ and a Plant‐Fed Asian Elephant Reveals an Unexpectedly High Diversity of Glycoside Hydrolase Family Enzymes.” PLoS One 9, no. 9: e106707. 25208077 10.1371/journal.pone.0106707 PMC4160196

[ece374333-bib-0016] Jin, Y. , J. Wang , Y. Yang , et al. 2023. “Impact of Land Use Change on Asian Elephant Habitat in Simao, Yunnan.” Forest Resources Management 3: 46–55.

[ece374333-bib-0017] Kambe, J. , Y. Sasaki , R. Inoue , et al. 2020. “Analysis of Infant Microbiota Composition and the Relationship With Breast Milk Components in the Asian Elephant ( *Elephas maximus* ) at the Zoo.” Journal of Veterinary Medical Science 82, no. 7: 983–989. 32350162 10.1292/jvms.20-0190 PMC7399312

[ece374333-bib-0018] Kantirakoon, S. , S. Klinhom , C. Kunasol , et al. 2026. “Characterization of Gut Microbiota in Wild Asian Elephants ( *Elephas maximus* ) From Five Forests Across Thailand.” Scientific Reports 16, no. 1: 27445. 42304055 10.1038/s41598-026-57801-x PMC13534693

[ece374333-bib-0019] Keady, M. M. , N. Prado , H. C. Lim , et al. 2021. “Clinical Health Issues, Reproductive Hormones, and Metabolic Hormones Associated With Gut Microbiome Structure in African and Asian Elephants.” Animal Microbiome 3, no. 1: 85. 34930501 10.1186/s42523-021-00146-9 PMC8686393

[ece374333-bib-0020] Klinhom, S. , C. Kunasol , S. Sriwichaiin , et al. 2025. “Characteristics of Gut Microbiota Profiles in Asian Elephants ( *Elephas maximus* ) With Gastrointestinal Disorders.” Scientific Reports 15, no. 1: 1327. 39779898 10.1038/s41598-025-85495-0 PMC11711614

[ece374333-bib-0021] Langille, M. G. I. , J. Zaneve , J. G. Caporaso , et al. 2013. “Predictive Functional Profiling of Microbial Communities Using 16S rRNA Marker Gene Sequences.” Nature Biotechnology 31, no. 9: 814–821. 10.1038/nbt.2676 PMC3819121 23975157

[ece374333-bib-0022] Lee, S. A. , S. Y. Kim , M. K. Sang , et al. 2024. “The Plant‐Associated Flavobacterium: A Hidden Helper for Improving Plant Health.” Plant Pathology Journal 40, no. 3: 251–260. 38835296 10.5423/PPJ.RW.01.2024.0019 PMC11162857

[ece374333-bib-0023] Ley, R. E. 2016. “Gut Microbiota in 2015: *Prevotella* in the Gut: Choose Carefully.” Nature Reviews Gastroenterology & Hepatology 13, no. 2: 69. 26828918 10.1038/nrgastro.2016.4

[ece374333-bib-0024] Li, W. W. 2017. “Risk Assessment of Human‐Elephant Conflict in Xishuangbanna.” Kunming: Southwest Forestry University.

[ece374333-bib-0025] Li, Y. J. 1998. “Current Status of Asian Elephants.” Wild 1: 22–23.

[ece374333-bib-0026] Lin, L. , X. M. Guo , A. D. Luo , et al. 2016. “Effects of Asian Elephant Feeding on Five Plant Species in the Wild Elephant Valley of Xishuangbanna.” Acta Theriologica Sinica 36, no. 2: 185–193.

[ece374333-bib-0028] Mohamed, M. M. A. , C. H. Myet , T. M. June , et al. 2021. “Anthropogenic Interferences Lead to Gut Microbiome Dysbiosis in Asian Elephants and May Alter Adaptation Processes to Surrounding Environments.” Scientific Reports 11, no. 1: 741. 33436882 10.1038/s41598-020-80537-1 PMC7803949

[ece374333-bib-0050] Niu, Y. L. , W. Liu , X. N. Fan , et al. 2023. “Beyond Cellulose: Pharmaceutical Potential for Bioactive Plant Polysaccharides in Treating Disease and Gut Dysbiosis.” Frontiers in Microbiology 14: 1183130. 37293228 10.3389/fmicb.2023.1183130 PMC10244522

[ece374333-bib-0029] Olivier, R. 1978. “Distribution and Status of the Asian Elephant.” Oryx 14, no. 4: 379–424.

[ece374333-bib-0030] Pappas, C. J. , C. Hamond , H. Pětrošová , and E. J. Putz . 2024. “Editorial: Spirochetal Diseases (Syphilis, Lyme Disease, and Leptospirosis): Transmission, Pathogenesis, Host‐Pathogen Interactions, Prevention, and Treatment.” Frontiers in Microbiology 15: 1510000. 39539710 10.3389/fmicb.2024.1510000 PMC11557550

[ece374333-bib-0031] Preez, D. L. L. , D. V. E. Walt , A. Valverde , et al. 2024. “A Metagenomic Survey of the Fecal Microbiome of the African Savanna Elephant ( *Loxodonta africana* ).” Animal Genetics 55, no. 4: 621–643. 38923598 10.1111/age.13458

[ece374333-bib-0032] Qin, L. 2007. “Habitat Selection of Asian Elephants ( *Elephas maximus* ) in Nangunhe Nature Reserve, Yunnan.” Beijing: Beijing Forestry University.

[ece374333-bib-0033] Rana, K. , N. Kentaro , N. NaomichiI , et al. 2020. “Comparison of the Fecal Microbiota of Two Monogastric Herbivorous and Five Omnivorous Mammals.” Animal Science Journal 91, no. 1: e13366. 32285557 10.1111/asj.13366 PMC7216987

[ece374333-bib-0051] Shin, N. , W. T. Whon , and J. Bae . 2015. “Proteobacteria: Microbial Signature of Dysbiosis in Gut Microbiota.” Trends in Biotechnology 33, no. 9: 496–503. 26210164 10.1016/j.tibtech.2015.06.011

[ece374333-bib-0036] Shoaib, M. , R. Bai , S. Li , Y. Xie , Y. Shen , and J. Ni . 2024. “Exploring the Diversity of Microbes and Natural Products From Fungus‐Growing Termite Tripartite Symbiosis.” Engineering Microbiology 4, no. 1: 100124. 39628791 10.1016/j.engmic.2023.100124 PMC11611000

[ece374333-bib-0037] Tan, A. J. , and L. J. Yu . 2015. “Distribution and Protection of Asian Elephants in China.” Protection Forest Science and Technology 5: 89–91.

[ece374333-bib-0038] Wang, N. , and L. Zhang . 2001. “Habitat Use by Asian Elephants in Simao During Dry Season and Conservation of Asian Elephants.” *Proceedings of the Symposium on Wildlife Ecology and Managementm, Guilin, 1*.

[ece374333-bib-0039] Wang, Y. , Y. Wang , J. Zhou , et al. 2024. “Exploring the Gut Microbiota of Healthy Captive Asian Elephants From Various Locations in Yunnan, China.” Frontiers in Microbiology 151, no. 403: 930–1403. 10.3389/fmicb.2024.1403930 PMC11468275 39397790

[ece374333-bib-0041] Wang, Z. H. , F. Chen , Z. C. Yang , et al. 2021. “Current Status and Prospects of Asian Elephant Research in China.” Forestry Construction 1: 6–11.

[ece374333-bib-0042] Wexler, H. M. 2007. “Bacteroides: The Good, the Bad, and the Nitty‐Gritty.” Clinical Microbiology Reviews 20, no. 4: 593–621. 17934076 10.1128/CMR.00008-07 PMC2176045

[ece374333-bib-0043] Yang, Z. Y. 2025. “Analysis of Gut Microbial Diversity and Investigation of Potential Pathogen Carriage in Wild Asian Elephants in Yunnan Province.” Kunming: Kunming University of Science and Technology.

[ece374333-bib-0048] Zhang, C. B. , J. M. Chen , Q. Wu , et al. 2023. “The Gut Microbiota of Young Asian Elephants With Different Milk‐Containing Diets.” Animals 13, no. 5: 916–916. 36899773 10.3390/ani13050916 PMC10000238

[ece374333-bib-0045] Zhang, C. , B. Xu , T. Lu , and Z. Huang . 2019. “Metagenomic Analysis of the Fecal Microbiomes of Wild Asian Elephants Reveals Microflora and Enzymes That Mainly Digest Hemicellulose.” Journal of Microbiology and Biotechnology 29, no. 8: 1255–1265. 31337187 10.4014/jmb.1904.04033

[ece374333-bib-0046] Zhang, L. , Y. N. Wang , and L. C. Ma . 2003. “Habitat Selection and Utilization by Asian Elephants in Simao, Yunnan.” Acta Theriologica Sinica 23, no. 3: 185–192.

[ece374333-bib-0047] Zhu, G. F. , X. Zheng , T. Lyu , et al. 2019. “Population Dynamics Analysis of Asian Elephants in Xishuangbanna‐Pu'er.” Forestry Construction 6: 85–90.

[ece374333-bib-0054] Zhu, J. Y. , T. Zheng , Y. W. Liu , et al. 2022. “Changes of Intestinal Mucosal Bacteria after Diarrhea in Mice Induced by High‐Fat and High‐Protein Diet.” Journal of Food and Nutrition Research 10, no. 2: 88–97.

