# Supplementary material for: Gut Microbiota Differences in Wild Asian Elephants From Pu'er, China
Source: Ecol Evol. 2026 Sep 8;16(9):e74333. doi: 10.1002/ece3.74333 (PMC13554142; doi:10.1002/ece3.74333)
Supplement: Supplementary file 1 [file ECE3-16-e74333-s001.pdf]

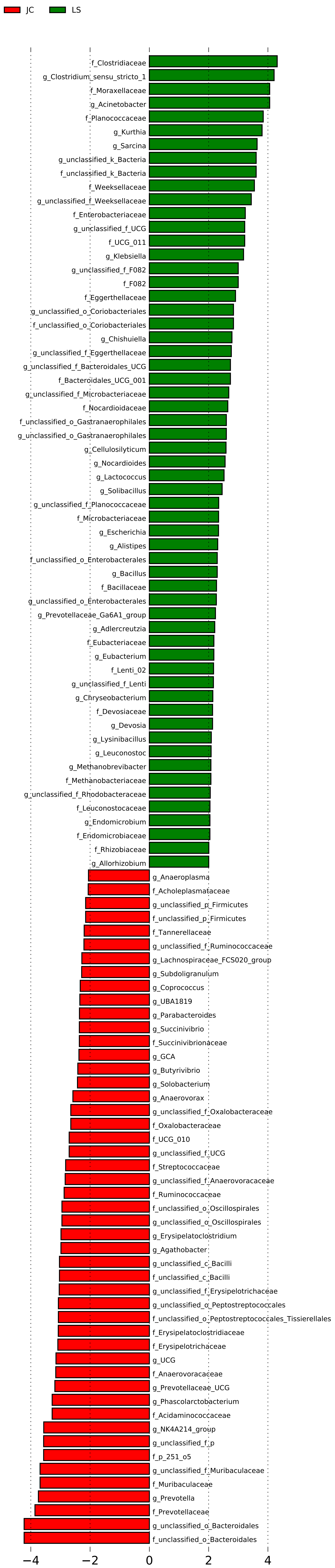

Fig. S1 Bar plot of LDA score distribution
